# Puree and Juice of Thai Mango and Pineapple Analyzed by High-Performance Thin-Layer Chromatography Hyphenated with Effect-Directed Assays

**DOI:** 10.3390/molecules26247683

**Published:** 2021-12-19

**Authors:** Gertrud E. Morlock, Newitchaya Wutthinithisanand, Doris Rauhut

**Affiliations:** 1 Chair of Food Science, Institute of Nutritional Science, and Interdisciplinary Research Center, Justus Liebig University Giessen, Heinrich-Buff-Ring 26-32, 35392 Giessen, Germany; neenee250@hotmail.com; 2 Department of Microbiology and Biochemistry, Hochschule Geisenheim University, Von-Lade-Str. 1, 65366 Geisenheim, Germany; Doris.Rauhut@hs-gm.de

**Keywords:** effect-directed analysis, healthy food, food processing, adulteration, authentication

## Abstract

The requirements for analytical tools are changing due to the global production chain, the increasing cases of adulteration, and the growing trend towards consumption of plant-based food products worldwide. The assessment of bioactivity of natural foods is currently not a quality criterion, and a paradigm shift is postulated. A non-targeted effect-directed profiling by high-performance thin-layer chromatography hyphenated with five different effect-directed assays was developed exemplarily for the puree and juice products of mango *Mangifera indica* L. (Anacardiaceae) and pineapple *Ananas comosus* (L.) Merr. (Bromeliaceae). Several bioactive compounds were detected in each sample. The additional bioactivity information obtained through effect-directed profiles improves, expands and modernizes product control. Non-target effect-directed profiling adds a new perspective to previous target analysis results that can be used not only to ensure health claims based on bioactive compounds, but also to detect unknown bioactive compounds coming from contamination or residues or changes caused by food processing.

## 1. Introduction

Bioactive compounds present in natural plant-based foods and their products attract increasingly both consumers and researchers. Epidemiological evidence indicates a contribution to the preventive effects in reducing the risk of certain common diseases, e.g., cardiovascular disorder, cancer, osteoporosis, infections, cataract and diabetes [1,2]. The spectrum and content of bioactive compounds in fruits vary, among other things, depending on the cultivar and maturity stage of the product [3,4]. The most prominent bioactive compounds found in tropical fruits are ascorbic acid (vitamin C), tocopherols (vitamin E), carotenoids (provitamin A candidates), flavonoids and thiol compounds. In particular, the widely distributed polyphenols are the predominant antioxidative secondary metabolite group [5].

The pineapple *Ananas comosus* (L.) Merr. (Bromeliaceae) and the mango *Mangifera indica* L. (Anacardiaceae) are important tropical fruits popular all over the world. Global pineapple and mango production in 2019 were about 28 million tons and 56 million tons, respectively, of which Thailand accounted for about 6% and 3% [6]. Pineapple is commonly consumed as fresh fruit, or processed to conventional products including canned fruit, jam, concentrated juice, and dried chips, or used as an ingredient in exotic food [7] or as material for pineapple wine production [8]. Thailand is not only a world leader in the export volume of canned pineapple, but many local cultivars are also unique in their taste and aroma [9]. Commercial cultivars of pineapple in Thailand are Pattavia, Phuket, Nanglae and Phulae. Pineapple is considered a very nutritious and functional fruit, as it is rich in vitamin A, vitamin B, and minerals such as calcium, phosphorus and iron [10]. It contains also several proteinases, such as bromelain, comosain and ananain, which support the digestive tract [11]. Moreover, it has several compounds with antioxidant properties, namely ascorbic acid, β-carotene and phenolic compounds such as flavonoids [12,13,14,15].

The mango fruit is widely accepted by consumers due to its succulence, sweet taste and exotic flavor. Mango flesh is consumed in both ripe and unripe stages. It is also processed to foods and drinks, such as pickled products, beverages, vinegar, chutneys, and desserts [16]. In Thailand, the major mango varieties, consumed fresh when the fruit is ripe, are Nam Dok Mai and Mahachanok. A small percentage of the production is processed to canned, dried and frozen mango [9]. Mangos contain bioactive compounds, such as ascorbic acid, carotenoids (e.g., β-carotene, violaxanthin, cryptoxanthin, neoxanthin, luteoxanthin and zeaxanthin), phenolic components, and mono-, di- and triterpenoids (e.g., ocimene, myrcene or limonene, terpinolene and careen) [2].

The domestic consumption and the export trade of pineapple and mango provide economic value to Thailand. Nevertheless, reports on bioactive compounds and antioxidant activities of these fruits from Thailand are rare. Since bioactive compounds generally occur as complex mixtures in a plant, it is a major challenge to identify the individual substances responsible for specific effects [17,18,19]. For such effect-directed profiling, high-performance thin-layer chromatography hyphenated with multi-imaging (UV/Vis/FLD) and effect-directed assays (EDA) was proven to be a good choice [20,21,22,23,24]. In this study, a non-target effect-directed profiling by HPTLC−UV/Vis/FLD−EDA was developed for puree and juice products of different Thai pineapple and mango cultivars to gain information on bioactive constituents. The obtained effect-directed profiles pointed to individual bioactive compounds and allowed a comparative evaluation with regard to their activity potential.

## 2. Results and Discussion

### 2.1. Physico-Chemical Characterization of the Produced Fruit Purees and Juices

Two cultivars each of mango and pineapple were chosen due to their importance as major commercial varieties and their widespread production in Thailand. The two pineapple cultivars Pattavia (PTV) and Nanglae (NL) were selected as sub-varieties of the smooth cayenne pineapple Pattavia variety. The two mango cultivars Nam Dok Mai (NDM) and Mahachanok (MHC) represent poly-embryonic and mono-embryonic ecotypes, respectively. At the commercial maturity stage, the fruits were harvested from the orchard at the Chiang Rai province in Northern Thailand. The regularity of fruit ripeness was controlled by the peel color observation in our study, i.e., 30–40% yellow for pineapple and fully yellow for mango. A puree and a juice sample were produced from each of the four cultivars. Thus, 8 fruit product samples were obtained. Their ripeness (content of total sugars and total acidity) and compositional values (content of total amino acids and total phenolics) were determined (Table 1). Total soluble solids, pH, and total acidity of pineapple and mango puree in both cultivars were in accordance with the literature [12,13,25,26,27,28,29]. The total sugar of pineapple was higher than that reported by Lu et al. [13]; however, values of natural plant-based food can vary depending on harvest time, climate influence each year, agricultural and soil management, among many other aspects. The sugar content normally increases during fruit ripening. 

### 2.2. Development of the Effect-Directed Profiling

**Figure 1:**
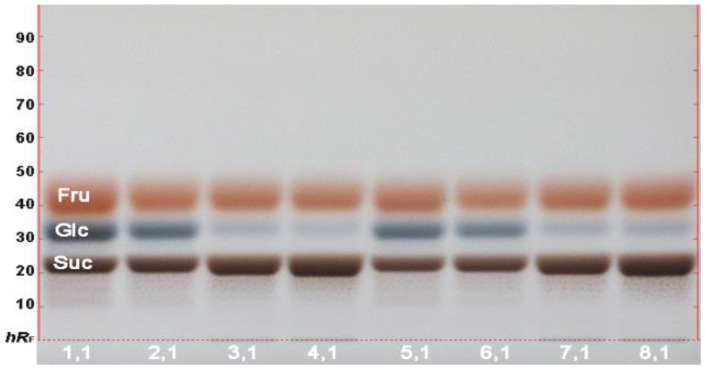
Saccharide analysis: HPTLC-Vis chromatogram of fructose (Fru), glucose (Glc) and sucrose (Suc) present in fruit product extracts (IDs 1–8, Table 2, 0.4 g/mL in methanol, 0.5 µL/area) on HPTLC plates silica gel 60 F_254_ with acetonitrile–water 4:1, *V*/*V*, plus 8 mg diphenylboric acid-2-aminoethylester (Table S1 from Supplementary Materials, mobile phase 6) derivatized with diphenylamine aniline *o*-phosphoric acid reagent and documented at white light illumination.

An effect-directed HPTLC method was developed on HPTLC plates silica gel 60 F_254_ (for the *B. subtilis* bioassay without F_254_). For a first impression, the more complex puree sample was chosen first. The methanolic extraction was performed at a solid-liquid ratio of 40%. A highly concentrated extract solution was preferred for the analysis of bioactive secondary metabolites, usually present in trace amounts. This allows the use of small sample volumes (rapid application) and easy adjustment to the different detection capabilities of biological and enzymatic assays (dilution step is faster than concentration step). Application as area (4 mg/area) was preferred due to the expected high load of sample matrix onto the adsorbent. Six different mobile phases (which resulted in successful separations in previous projects) were investigated (Table S1 from Supplementary Materials). Solvent mixture 1 consisting of toluene–ethyl acetate–methanol–formic acid 2.4:1.8:0.7:0.06, *v*/*v*/*v*/*v*, was suited best, as it distributed the sample components well along the developing distance, especially detectable after derivatization with the anisaldehyde sulfuric acid reagent at FLD 366 nm. A large portion of each methanolic extract remained in the initial start zone area, to which the sugar content contributed most, being as high as 16% in the 8 fruit product samples (Table 1). The individual saccharides of the samples, applied at 200 µg/area, were determined using a more polar mobile phase for separation (Table S1 from Supplementary Materials), mobile phase 6: acetonitrile–water 4:1, *v*/*v*, plus 8 mg diphenylboric acid-2-aminoethylester) and the diphenylamine aniline *o*-phosphoric acid reagent for detection at white light illumination. The 8 saccharide profiles showed glucose, fructose and sucrose being present (Figure 1). Among these, the mango cultivars (Table 2, puree IDs 3,1 and 4,1; juice IDs 7,1 and 8,1) showed a lower glucose and instead a higher sucrose content than the pineapple cultivars (puree IDs 1,1 and 2,1; juice IDs 5,1 and 6,1).

### 2.3. Selection of the Extraction Solvent for Effect-Directed Profiling

**Figure 2:**
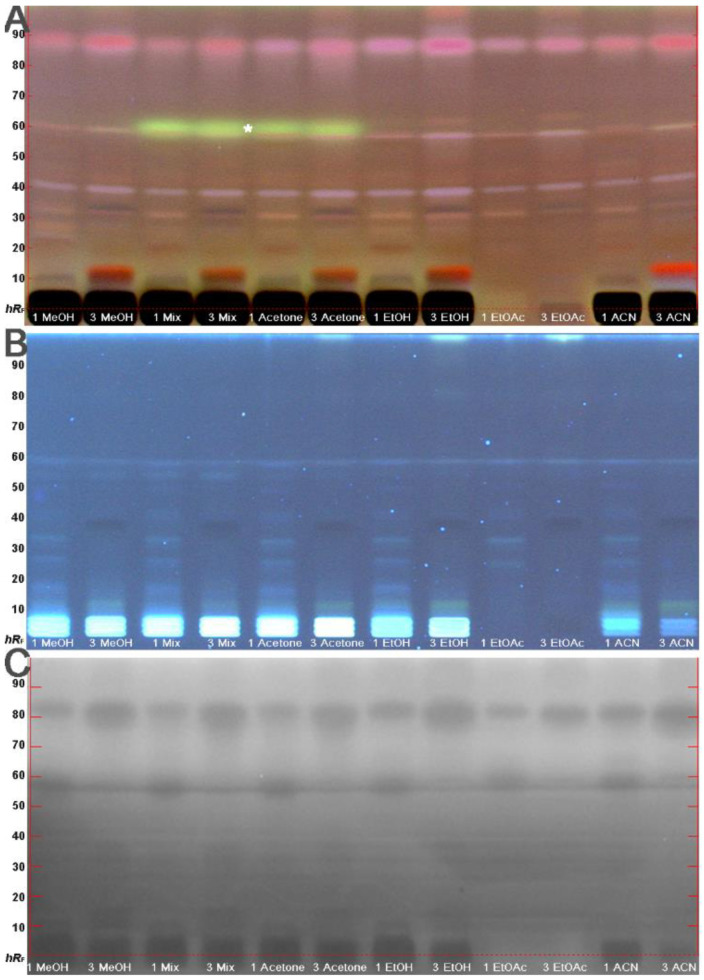
Investigation of different solvent selectivities for extraction: HPTLC chromatogram at FLD 366 nm after derivatization with anisaldehyde sulfuric acid reagent (**A**), at FLD 366 nm before the bioassay (**B**) and bioautogram after *Aliivibrio fischeri* bioassay, depicting bioluminescence after 30 min as grey scale image (**C**) of methanol (MeOH), mixture of methanol/acetone 1:1 (Mix), acetone, ethanol (EtOH), ethyl acetate (EtOAc) and acetonitrile (ACN) extracts of puree sample IDs 1 and 3 (Table 2, 0.4 g/mL) applied as area (6.0 × 3.0 mm, 10 µL/area), developed on HPTLC plates silica gel 60 F_254_ with toluene–ethyl acetate–methanol–formic acid 2.4:1.8:0.7:0.06, *v*/*v*/*v*/*v*, in twin-trough chamber 20 × 10 cm (* yellow acetone-soluble conta mination from plastic flask material).

Sample preparation was performed as minimally as possible to minimize any influence (alteration) in the fruit products produced. Different extraction solvents (i.e., methanol, acetone, methanol/acetone 1:1, ethanol, ethyl acetate, and acetonitrile) were comparatively studied for the intended non-targeted effect-directed profiling (Figure 2, all 4 mg/area). For polar extraction solvents, the co-extracted saccharides caused a high matrix loading on the start zone area of the adsorbent. This limited not only the amount of sample that could be applied, but also the development with more polar mobile phases (Table S1 from Supplementary Materials, mobile phases 4 and 6). In comparison, the ethyl acetate extracts discriminated the co-extraction of saccharides, as evident in the absent dark start zone area. This was found to be advantageous, in case higher sample amounts need to be used depending on the assay response. In addition, the ethyl acetate extracts contained a similar pattern of medium polar components that was almost comparable to that of the other solvents. Therefore, to cover different selectivities, not only methanol but also ethyl acetate were chosen as extraction solvent (Table 2). Apart from UV/Vis/FLD detections and the use of derivatization reagents, the compounds in the extracts were detected via the Gram-negative *Aliivibrio fischeri* bioassay (Figure 2C). This assay detects more universally any bioactive chemicals interfering with the energetic metabolism of the bacteria and is commonly used in environmental research [30]. The bioautogram revealed a similar pattern of bioactive compounds within the different samples. As the bioactivity pattern of the applied 10 µL sample (4 mg/area) was faint, it was evident that a higher sample amount was required to detect the bioactive components present at low concentrations. Therefore, a 5-fold amount of each fruit product extract (solid-liquid extract ratio of 20% in ethyl acetate) was applied (100 µL/area) in the following.

### 2.4. Physico-Chemical Profiling of the Fruit Product Extracts

**Figure 3:**
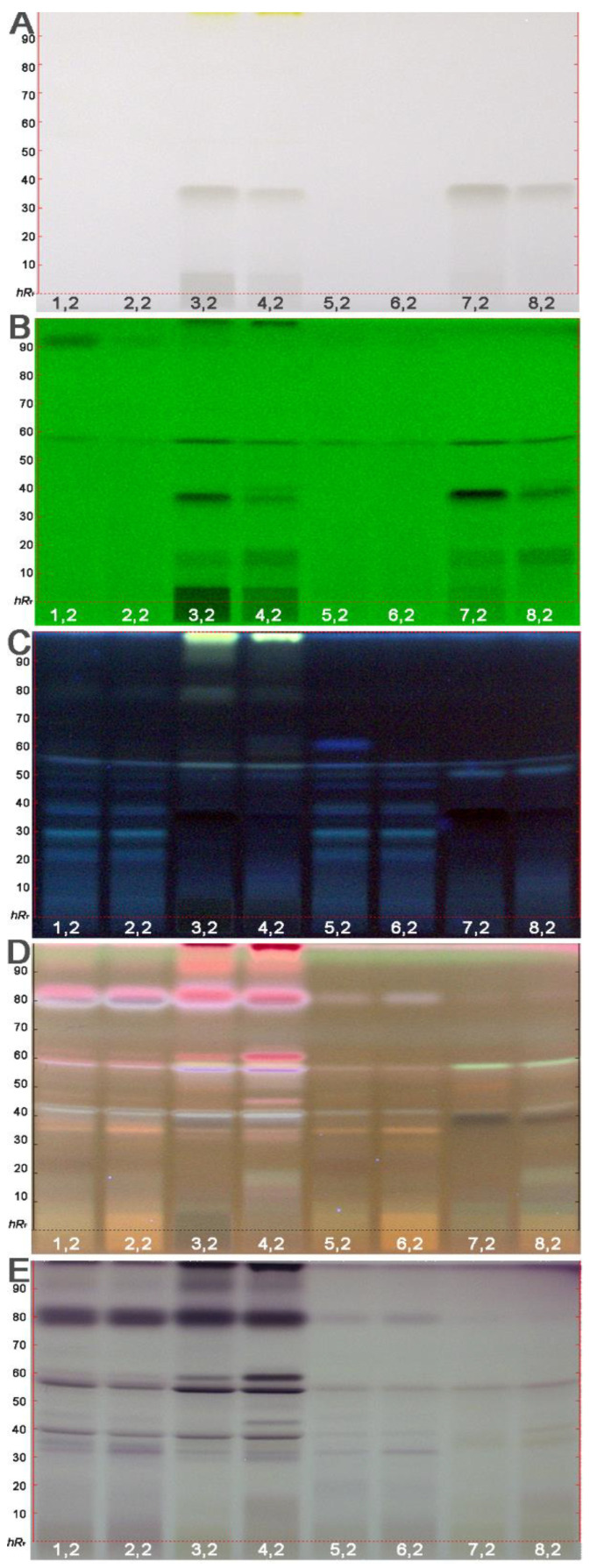
Physico-chemical profiling: HPTLC chromatograms at white light illumination (Vis, **A**), UV 254 nm (**B**), FLD 366 nm (**C**) and after derivatization with anisaldehyde sulfuric acid reagent (**D** FLD 366 nm and **E** Vis) of the puree and juice extracts (IDs 1–8, Table 2, 0.2 g/mL in ethyl acetate, 100 µL/area of 8.0 × 6.0 mm) developed on HPTLC plates silica gel 60 F_254_ with toluene–ethyl acetate–methanol–formic acid 2.4:1.8:0.7:0.06, *v*/*v*/*v*/*v*, in twin-trough chamber 10 cm × 10 cm.

The physico-chemical profiling of the ethyl acetate extracts of the 8 different fruit products provided information on the absorbance or fluorescence of individual compounds. Close to the solvent front of the separated mango puree extracts (IDs 3,2 and 4,2), a yellow zone, most likely carotenoids, was visible (Figure 3A). UV-active compounds were comparatively more pronounced in the mango (IDs 3,2; 4,2; 7,2 and 8,2) than pineapple product extracts (Figure 3B). Among the mango products, the puree extracts (IDs 3,2 and 4,2) contained more UV-active compounds than the juice extracts, mainly retained in the start area of the adsorbent. 

In the FLD 366 nm chromatogram (Figure 3C), a dominantly yellow fluorescent zone was observed in the solvent front of the mango puree extracts (IDs 3,2 and 4,2). This zone was also visible and UV-active as mentioned. Tentatively, more blue fluorescent zones were observed in the pineapple (IDs 1,2; 2,2; 5,2 and 6,2) than mango products (IDs 3,2; 4,2; 7,2 and 8,2). After derivatization with the anisaldehyde sulfuric acid reagent and detection at FLD 366 nm (Figure 3D) or with white light illumination (Figure 3E), further components of the fruit products were detectable. For example, a strongly pink fluorescent (Figure 3D) or lilac (Figure 3E) compound at *hR*_F_ 80 was detected only by chemical derivatization, proving that the commonly used UV/Vis/FLD detection is not sufficient for quality control. In particular, this compound zone also proved to have multiple bioactivities, as discussed subsequently. As expected by the natural density of the product and verified by all the different detection modes, the puree extract samples (IDs 1,2–4,2) were more complex and rich in natural compounds.

### 2.5. Effect-Directed Profiling of the Fruit Product Extracts

**Figure 4:**
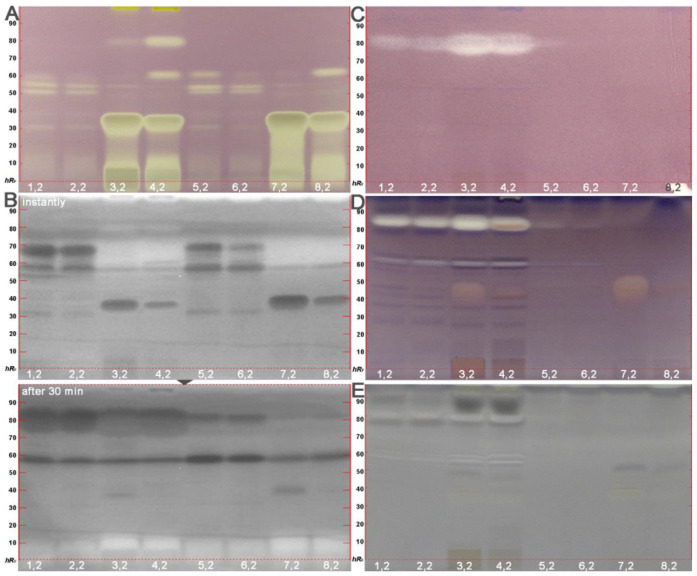
Effect-directed profiling: HPTLC autograms after DPPH• assay (**A**), *Aliivibrio fischeri* bioassay, instantly and after 30 min (**B**), *Bacillus subtilis* bioassay (**C**), AChE (**D,** here zones shifted upwards by 2 mm) and tyrosinase inhibition assays I detected at white light illumination (except for B: bioluminescence depicted as grey scale image) of puree and juice extracts (IDs 1–8, Table 2, 0.2 g/mL in ethyl acetate, 100 µL/area of 8.0 × 6.0 mm) developed on HPTLC plates silica gel 60 F_254_ with toluene–ethyl acetate–methanol–formic acid 2.4:1.8:0.7:0.06, *v*/*v*/*v*/*v*, in twin-trough chamber 10 cm × 10 cm (chromatograms at UV 254 nm and FLD 366 nm before the respective assay detection in Table S2 from Supplementary Materials).

The effect-directed profiling of the ethyl acetate extracts of the 8 different fruit products provided information on bioactive components using five different effect-directed assays (i.e., one chemical, two biological and two enzymatic assays). The DPPH• radical scavenging assay (Figure 4A) showed for both mango products (puree and juice) the strongest radical scavenging (antioxidative) compound zone at the start area and at *hR*_F_ 37 due to the ascorbic acid addition. This addition was required during juice production to prevent the enzymatic browning reaction, and thus, darkening of mango products.

Nevertheless, clear differences in the antioxidative response at *hR*_F_ 60 and *hR*_F_ 80 were evident for the two mango cultivars (IDs 3,2 versus 4,2; 7,2 versus 8,2). The comparison of the two mango products also revealed a difference. The enzymatic or heat treatment during juice production degraded the antioxidative compound zone at *hR*_F_ 80 (e.g., IDs 4,2 versus 8,2).

At the same position (*hR*_F_ 37) as the previously mentioned main antioxidative compound zone (ascorbic acid), the Gram-negative *Aliivibrio fischeri* bioautogram (Figure 4B) revealed a dominant bioactive dark zone in both mango products and cultivars which, however, substantially decreased over the 30 min imaging inspection. This indicated an acute transient effect, i.e., the energetic metabolism of the bacteria was immediately but temporarily disrupted by this compound and recovered almost completely after 30 min. In the pineapple cultivars, two further dominant antimicrobial zones at *hR*_F_ 58 and 68 were observed. The similar acute transient effect was observed for the antimicrobial zone at *hR*_F_ 68, whereas the antimicrobial zone at *hR*_F_ 58 increased in activity over the monitored 30 min period. The latter indicated a lasting effect with a delayed increase in the activity response. Over the monitoring period, a new antimicrobial compound appeared at *hR*_F_ 80 mainly in both fruit purees, as evident in the 30-min bioautogram. This indicated a delayed increase in the activity response.

The Gram-positive *Bacillus subtilis* bioassay (Figure 4C) revealed the same antimicrobial compound zone at *hR*_F_ 80 in both fruit puree extracts. This colorless zone at *hR*_F_ 80 acting against *B. subtilis* was also the main inhibitor of the AChE (Figure 4D, IDs 1,2–4,2). Another weaker AChE inhibiting zone was detected at *hR*_F_ 60. Both colorless zones were almost absent in the juice extracts, most likely degraded by the enzymatic or heat treatment during the juice production. Especially, the mango cultivar NDM (IDs 3,2 and 7,2) revealed a further AChE inhibiting compound zone at *hR*_F_ 45. 

The tyrosinase inhibition assay (Figure 4E) showed three colorless inhibition zones in the puree extracts (IDs 1,2–4,2) with the main inhibition zone at *hR*_F_ 80. This multipotent compound zone showed already a pronounced activity against *B. subtilis* and AChE. Vice versa, the dark zone above (*hR*_F_ 85) in the mango puree extracts (ID 3,2 and 4,2) triggered the fruit browning. Another weaker browning-promoting dark zone at *hR*_F_ 78 was evident in all puree extracts (IDs 1,2–4,2). 

Differences in the activity profiles depending on the fruit processing were evident. Both purees contained a multipotent zone at *hR*_F_ 80 (active against both bacteria, AChE and tyrosinase, Figure 4B–E), which was lost during the production of juice made out of the puree. This was a more general observation across all the different assays, as the more apolar bioactive compounds (evident in the upper autogram part) were partially or totally lost during the production of juice. Although it was expected that puree is richer in bioactive compounds than juice, the severe loss was surprising. All in all, the bioprofiling of food products turned out to be a rich source of information with regard to changes in the activity potential caused by food processing.

## 3. Materials and Methods

### 3.1. Chemicals and Reagents

Bi-distilled water was produced using a Heraeus Destamat Bi-18E (Thermo Fisher Scientific, Schwerte, Germany). Ethyl acetate (≥99.7%) and ammonia (25%) were obtained from Th. Geyer, Renningen, Germany. All amino acids, lithium citrate, all mineral salts for producing phosphate-buffered saline (PBS), acetone (≥99.5%), acetonitrile (≥99.9%), diphenylamine, aniline, 2,2-diphenyl-1-picrylhydrazyl radical (DPPH•, 97%), acetylcholinesterase (AChE) from *Electrophorus electricus* (≥245 U/mg, 10 kU/vial), bovine serum albumin (BSA, fraction V, ≥98%) and tyrosinase from mushroom (≥1000 U/mg, 25 kU/vial) were obtained from Fluka Sigma-Aldrich, Steinheim, Germany. 1-Naphthyl acetate (≥98%) and 2-naphthyl-α-D-glucopyranoside were obtained by AppliChem, Darmstadt, Germany. Toluene, ethanol, 2-propanol (all ≥ 99.9%), formic acid (≥98%), sulfuric acid (96%), diphenylboric acid-2-aminoethylester, 4-methoxybenzaldehyde (anisaldehyde, ≥97.5%), tris(hydroxymethyl)amino¬methane (TRIS, ≥99.9%) and thiazol blue tetrazolium bromide (3-(4,5-dimethylthiazol-2-yl)-2,5-diphenyl-tetrazolium bromide, MTT) were purchased from Carl Roth, Karlsruhe, Germany. Methanol (>99.8%) was purchased from VWR, Darmstadt, Germany. (2S)-2-Amino-3-(3,4-dihydroxyphenyl) propionic acid (levodopa) was obtained from Santa Cruz Biotechnology, Dallas, TX, USA. 1-Butanol was from Alfa Aesar, Karlsruhe, Germany. HPTLC plates silica gel 60 F_254_ (without F_254_ for *B. subtilis* bioassay), 20 cm × 10 cm, ascorbic acid, potassium disulfite, sodium hydroxide, Gram-positive soil bacteria *Bacillus subtilis* subsp. spizizenii (DSM-618), and Folin-Ciocalteu’s phenol reagent were from Merck, Darmstadt, Germany. Gram-negative marine *A. fischeri* bacteria (NRRL-B11177, strain 7151) were bought from the Leibniz Institute DSMZ (German Collection of Microorganisms and Cell Cultures), Berlin, Germany. Fruit samples were from the orchard at Chiang Rai province, Northern Thailand.

### 3.2. Production of Puree Samples

Ripe pineapples were washed with water, peeled, cut into small pieces and crushed to form pineapple puree. Then 50 mg/L of sulfur dioxide was added. Ripe mangoes were also washed, peeled, cut and crushed, but after peeling the seeds were removed. The mango purees were treated with 40 mg/L of ascorbic acid and 50 mg/L of sulfur dioxide. Each fruit puree (1 kg) was placed in a polyethylene pouch and stored at −18 °C until further use. 

### 3.3. Production of Juice Samples

The frozen puree of each cultivar was thawed at ambient temperature and treated with pectinase (400 μL/L; Trenolin^®^Super DF, Erbslöh, Geisenheim, Germany). The puree was incubated at 45–50 °C for 1 h. Then, it was heated at 70 °C for 5 min to inactivate the enzymes. The juice was separated from the solid material part by centrifugation at 5000× *g* at 4 °C for 10 min. Juice (100 mL) was filled in a polyethylene bottle and stored at −18 °C until use. 

### 3.4. Physico-Chemical Characterization of the Produced Purees and Juices

The standard parameters were established as follows: total soluble solids (TSS) was measured by a digital automatic refractometer (Abbemat, Anton Paar^®^, St. Albans, Austria). The total saccharide content was enzymatically determined using a Konelab 20XTi analyzer (Thermo Fisher Scientific, Schwerte, Germany) and its proper kits (EnzytecTM fluid, Thermo Fisher Scientific). Total acidity and pH were analyzed by a Schott titrator (Titroline alpha plus, SI-Analytics, Texas City, TX, USA). The content of total phenols was evaluated by the Folin assay (Singleton and Rossi 1965) using an automatic analyzer (Konelab 20XTi, Thermo Fisher Scientific). Amino acids were determined by an Amino Acid Analyzer S433 (Sykam GmbH, Eresing, Germany). All measurements were made in triplicate.

### 3.5. HPTLC−UV/Vis/FLD−EDA Analysis of the Produced Purees and Juices

#### 3.5.1. Extraction

For the selection of the extraction solvent, 400 mg each of pineapple (PTW) or mango (NDM) puree were placed in a 1.5 mL centrifuge tube, mixed and extracted with 1 mL solvent (methanol, acetone, ethanol, ethyl acetate, and acetonitrile) in the ultrasonic bath for 10 min. Each suspension was centrifuged (5 min, 11,600× *g*) and the supernatant transferred into an autosampler vial (1.8 mL). For effect-directed profiling, the puree (2 g) and juice samples (2 mL) were extracted with 10 mL ethyl acetate, mixed (vortexed) and centrifuged (5 min, 3000× *g*). The supernatant was removed with a Pasteur pipette, was transferred to a 10 mL glass vial and stored at −18 °C until use.

#### 3.5.2. Sample Application and Development

Instrumentation was used from CAMAG. Plates were cut to smaller pieces using the smartCUT Plate Cutter. Solutions were sprayed as a band (8 mm) for sugar analysis or as area (6.0 × 3.0 mm for 10 µL or 8.0 × 6.0 mm for 100 µL) for other analyses on HPTLC plates with the Automatic TLC Sampler (ATS) 4. Up to 8 tracks were applied onto an HPTLC plate, 10 cm × 10 cm, with a distance of 10 mm from the lower edge, 15 mm distance from the left edge and 10 mm distance between the bands. Sample volumes ranged 10 µL for extraction solvent selection and 100 µL for effect-directed profiling. Development was performed with a mixture of with toluene–ethyl acetate–methanol–formic acid 2.4:1.8:0.7:0.06, *v*/*v*/*v*/*v* [31] up to a migration distance of 60 mm from the lower plate edge in a twin-through chamber. The chromatogram was dried in a stream of warm air (hair dryer) for 2 min.

#### 3.5.3. Derivatization and Documentation

For derivatization, the HPTLC plate was immersed into the anisaldehyde sulfuric acid reagent (5 mL concentrated sulfuric acid was carefully added to a mixture of 500 µL anisaldehyde, 10 mL acetic acid, and 100 mL methanol) or diphenylamine aniline *o*-phosphoric acid reagent (mixture of 70 mL aniline solution, 70 mL diphenylamine solution, 2% each in acetone, and 10 mL *o*-phosphoric acid, 85%) using the TLC Chromatogram Immersion Device. The immersion speed was 3 cm/s and the immersion time 2 s. The plates were heated at 110 °C on the TLC Plate Heater for 5 min. The HPTLC chromatograms were documented at UV 254, FLD 366 nm and white light illumination (reflection and transmission mode) using the TLC Visualizer. All data obtained was processed with the software winCATS, version 1.4.7.2018.

#### 3.5.4. Effect-Directed Detection

The HPTLC plates were immersed into the respective assays using the TLC Chromatogram Immersion Device (immersion speed 3 cm/s), heated using the TLC Plate Heater, and if not stated otherwise, documented using the TLC Visualizer or DigiStore 2 Documentation System. Before application of the biological assays, the chromatogram was neutralized with ammonia vapor for 5 min and freed from any excess vapor or acidic mobile phase traces (Automatic Developing Chamber 2; relative humidity control glass flask filled with dry molecular sieve) for 25 min.

Radical scavenging assay: The chromatogram was dipped in a 0.02% methanolic DPPH• solution [32] for 5 s (immersion time). The chromatogram was dried in the dark at room temperature for 90 s, heated at 60 °C for 30 s and documented at white light illumination (reflectance mode), repeated after a day.

*A. fischeri* bioassay: The nutrient medium for the bacterial suspension was prepared as described [33]. The dried, neutralized chromatogram was immersed into the suspension for 3 s. Bioluminescence images (bioautograms) were recorded and processed with the BioLuminizer software, version 1.0.2.6107. Ten images were recorded over 30 min at time intervals of 3 min, each over an exposure time of 50 s.

*B. subtilis* bioassay: The dried, neutralized chromatogram was immersed in the bacterial suspension for 5 s and incubated at 37 °C for 2 h [31,34]. For visualization, the plates were immersed into a 0.2% PBS-buffered MTT solution for 1 s. During the incubation for 30 min at 37 °C, the MTT was reduced to a purple formazan dye, stopped by drying the plate at 50 °C for 5 min. Bioautograms were documented at white light illumination (reflection mode).

AChE inhibition assay: The dried, neutralized plate was immersed in the enzyme solution (AChE 666 units and 100 mg BSA in 100 mL 0.05 M TRIS buffer, pH 7.8) for 5 s. The plate was incubated for 25 min at 37 °C according to Akkad and Schwack [35]; Hage and Morlock [36]. For visualization, the chromatogram was immersed in the substrate solution (25 mg α-naphthyl acetate and 50 mg Fast Blue salt B in 90 mL ethanol-water, 1:2) and documented at white light illumination (reflectance mode). 

Tyrosinase inhibition assay: The dried, neutralized plate was immersed in the enzyme solution (400 U/mL mushroom tyrosinase in 0.02 M phosphate buffer, pH 6.8) for 5 s [37]. The plate was dried for about 2 min and immersed in the substrate solution (L-DOPA, 18 mM in phosphate buffer, pH 6.8) for 3 s, followed by incubation for 15 min at room temperature. The dried plate was recorded at white light illumination (reflectance mode).

## 4. Conclusions

The bioactivity patterns revealed multiple bioactive compounds of mango and pineapple purees and juices, self-produced from self-harvested authentic fruits. The bioactivity assessment can be used to valorize and add value to the plant-based products. It can also be used with regard to distinct health claims, e.g., based on antioxidative compounds. Some of the bioactive compounds were not detected by UV/Vis/FLD, but first with the planar biochemical or biological assays. Effect-directed profiling thus makes product control more powerful. Since it is a non-targeted method, also unknown effective compounds, which are not in the focus of current analysis, can be detected. This is of high importance due to the global food processing chain. Compared to the status quo, product changes caused by contamination, adulteration, processing, treatments, etc. can be more comprehensively and easily detected through multi-imaging exploiting an array of different detection techniques.

## Figures and Tables

**Table 1:** Properties of the produced purees and juices of the pineapple (PTV and NL) and mango cultivars (NDM and MHC). Each value shows the mean ± standard deviation from three measurement replicates. Different letters (a–d) derived from the ANOVA and DMRT test indicate significant differences among samples.

	Pineapple Cultivar		Mango Cultivar	
	PTV	NL	NDM	MHC
Parameters	Produced Puree			
Total Soluble Solids (°Brix)	14.4 ± 0.2 ^d^	15.3 ± 0.1 ^c^	18.0 ± 0.2 ^a^	16.4 ± 0.1 ^b^
Total Sugar (g/L)	133 ± 0 ^d^	142 ± 0 ^c^	159 ± 0 ^a^	151 ± 0 ^b^
pH	4.6 ± 0.0 ^a^	4.5 ± 0.0 ^a^	3.7 ± 0.0 ^c^	4.0 ± 0.0 ^b^
Total Acidity (pH 8.1 as citric acid, g/L)	2.4 ± 0.0 ^c^	2.4 ± 0.0 ^c^	7.8 ± 0.2 ^a^	4.1 ± 0.1 ^b^
	Produced Juice			
Total Amino Acids (mg/L)	3335 ± 8 ^a^	2790 ± 11 ^b^	1641 ± 4 ^c^	1366 ± 14 ^d^
Total Phenolics (mg/L)	426 ± 3 ^d^	442 ± 4 ^c^	845 ± 6 ^a^	616 ± 4 ^b^

**Table 2:** Produced fruit products (IDs 1–8) extracted either with methanol (x,1) or ethyl acetate (x,2).

Puree Extract		Juice Extract	
ID	Fruit Cultivar	ID	Fruit Cultivar
1,1	Pineapple PTV	5,1	Pineapple PTV
1,2		5,2	
2,1	Pineapple NL	6,1	Pineapple NL
2,2		6,2	
3,1	Mango NDM	7,1	Mango NDM
3,2		7,2	
4,1	Mango MHC	8,1	Mango MHC
4,2		8,2	

## Data Availability

Data are available from the authors on request.

## Supplementary Materials

The following are available online, Table S1: Investigated mobile phases 1–6, Table S2: Chromatograms at UV 254 nm and FLD 366 nm before the respective assay detection.

## Sample Availability

Samples of the compounds are not available from the authors.

## References

[1] Block G., Patterson B., Subar A. (1992). Fruit, vegetables, and cancer prevention: A review of the epidemiological evidence. Nutr. Cancer.

[2] Ribeiro S.M.R., Schieber A., Watson R.R., Preedy V.R. (2010). Chapter 34-Bioactive Compounds in Mango (*Mangifera indica* L.). Bioactive Foods in Promoting Health: Fruits and Vegetables.

[3] Giuffrè A.M. (2019). Bergamot (*Citrus bergamia*, Risso): The Effects of Cultivar and Harvest Date on Functional Properties of Juice and Cloudy Juice. Antioxidants.

[4] Siriwoharn T., Wrolstad R.E., Finn C.E., Pereira C.B. (2004). Influence of cultivar, maturity, and sampling on blackberry (*Rubus,* L. Hybrids) anthocyanins, polyphenolics, and antioxidant properties. J. Agric. Food Chem..

[5] González-Aguilar G., Robles-Sánchez R.M., Martínez-Téllez M.A., Olivas G.I., Alvarez-Parrilla E., Rosa L.A. (2008). Bioactive compounds in fruits: Health benefits and effect of storage conditions. Stewart Postharvest Rev..

[6] Food and Agricultural Organization of the United Nations. http://www.fao.org/faostat/en/#data/QC.

[7] Barretto L.C.d.O., Moreira J.d.J.d.S., Santos J.A.B.d., Narendra N., Santos R.A.R.d. (2013). Characterization and extraction of volatile compounds from pineapple (*Ananas comosus* L. Merril) processing residues. Food Sci. Technol (Camp.).

[8] Pino J.A., Queris O. (2010). Analysis of volatile compounds of pineapple wine using solid-phase microextraction techniques. Food Chem..

[9] Chomchalow N., Somsri S., Na Songkhla P. (2008). Marketing and Export of Major Tropical Fruits from Thailand. AU J.T..

[10] Gardner P.T., White T.A.C., McPhail D.B., Duthie G.G. (2000). The relative contributions of vitamin C, carotenoids and phenolics to the antioxidant potential of fruit juices. Food Chem..

[11] Mhatre M., Tilak-Jain J., De S., Devasagayam T.P.A. (2009). Evaluation of the antioxidant activity of non-transformed and transformed pineapple: A comparative study. Food Chem. Toxicol..

[12] Kongsuwan A., Suthiluk P., Theppakorn T., Srilaong V., Setha S. (2009). Bioactive compounds and antioxidant capacity of phulae and nanglae pineapple. As. J. Food Ag-Ind..

[13] Lu X.-H., Sun D.-Q., Wu Q.-S., Liu S.-H., Sun G.-M. (2014). Physico-chemical properties, antioxidant activity and mineral contents of pineapple genotypes grown in china. Molecules.

[14] Freitas A., Moldão-Martins M., Costa H.S., Albuquerque T.G., Valente A., Sanches-Silva A. (2015). Effect of UV-C radiation on bioactive compounds of pineapple (*Ananas comosus* L. Merr.) by-products. J. Sci. Food Agric..

[15] Ferreira E.A., Siqueira H.E., Boas E.V.V., Hermes V.S., Rios A.D.O. (2016). Bioactive compounds and antioxidant activity of pineapple fruit of different cultivars. Rev. Bras. Frutic..

[16] Ramteke R.S., Vilajayalaskshmi M.R., Eipeson W.E. (1999). Processing and value addition to mangoes. Indian Food Ind..

[17] Morlock G.E., Schwack W. (2010). Hyphenations in planar chromatography. J. Chromatogr. A.

[18] Morlock G.E. (2014). Background mass signals in TLC/HPTLC-ESI-MS and practical advices for use of the TLC-MS interface. J. Liq. Chromatogr. Relat..

[19] Morlock G.E., Worsfold P.J., Poole A., Townshend A., Miro M. (2019). Bioassays–Effects-detection in chromatography. Reference Module in Encyclopedia of Analytical Science.

[20] Krüger S., Urmann O., Morlock G.E. (2013). Development of a planar chromatographic method for quantitation of anthocyanes in pomace, feed, juice and wine. J. Chromatogr. A.

[21] Cretu G.C., Morlock G.E. (2014). Analysis of anthocyanins in powdered berry extracts by planar chromatography linked with bioassay and mass spectrometry. Food Chem..

[22] Morlock G.E., Klingelhöfer I. (2014). Liquid chromatography-bioassay-mass spectrometry for profiling of physiologically active food. Anal. Chem..

[23] Teh S.-S., Morlock G. (2015). Analysis of bioactive components of oilseed cakes by high-performance thin-layer chromatography-(bio)assay combined with mass spectrometry. Chromatography.

[24] Ristivojević P.M., Morlock G.E. (2018). Effect-directed classification of biological, biochemical and chemical profiles of 50 German beers. Food Chem..

[25] Joomwong A., Sornsrivichai J. (2005). Morphological characteristic, chemical composition and sensory quality of pineapple fruit in different seasons. Chiang Mai Univ. J. Nat. Sci..

[26] Nilprapruck P., Pradisthakarn N., Authanithee F., Keebjan P. (2008). Effect of exogenous methyl jasmonate on chilling injury and quality of pineapple (*Ananas comosus* L.) cv. Pattavia. SUSTJ.

[27] Boonyaritthongchai P., Puthmee T., Wongs-Aree C. (2015). Quality changes of fresh-cut ‘Mahachanok’ mango at different storage temperatures. Acta Hortic..

[28] Lapcharoensuk R., Phannote N., Kasetyangyunsapa D. Physicochemical properties of pineapple at difference maturity. Proceedings of the Conference paper of the 10th TSAE International Conference.

[29] Chuensombat N., Rungraeng N., Setha S., Suthiluk P. (2019). A preliminary study of high pressure processing effect on quality changes in ‘Nanglae’ pineapple juice during cold storage. JFAT.

[30] Logemann A., Schafberg M., Brockmeyer B. (2019). Using the HPTLC-bioluminescence bacteria assay for the determination of acute toxicities in marine sediments and its eligibility as a monitoring assessment tool. Chemosphere.

[31] Jamshidi-Aidji M., Morlock G.E. (2016). From bioprofiling and characterization to bioquantification of natural antibiotics by direct bioautography linked to high-resolution mass spectrometry: Exemplarily shown for *Salvia miltiorrhiza* root. Anal. Chem..

[32] Pozharitskaya O.N., Ivanova S.A., Shikov A.N., Makarov V.G. (2008). Separation and free radical-scavenging activity of major curcuminoids of *Curcuma longa* using HPTLC-DPPH method. Phytochem. Anal..

[33] (2009). Water Quality-Determination of the Inhibitory Effect of Water Samples on the Light Emission of Vibrio Fischeri (Luminescent Bacteria Test)-Part 1: Method Using Freshly Prepared Bacteria (ISO 11348-1:2007).

[34] Jamshidi-Aidji M., Morlock G.E. (2015). Bioprofiling of unknown antibiotics in herbal extracts: Development of a streamlined direct bioautography using *Bacillus subtilis* linked to mass spectrometry. J. Chromatogr. A.

[35] Akkad R., Schwack W. (2010). Multi-enzyme inhibition assay for the detection of insecticidal organophosphates and carbamates by high-performance thin-layer chromatography applied to determine enzyme inhibition factors and residues in juice and water samples. J. Chromatogr. B.

[36] Hage S., Morlock G.E. (2017). Bioprofiling of Salicaceae bud extracts through high-performance thin-layer chromatography hyphenated to biochemical, microbiological and chemical detections. J. Chromatogr. A.

[37] Krüger S., Bergin A., Morlock G.E. (2018). Effect-directed analysis of ginger (*Zingiber officinale*) and its food products, and quantification of bioactive compounds via high-performance thin-layer chromatography and mass spectrometry. Food Chem..

