# Supplementary material for: Puree and Juice of Thai Mango and Pineapple Analyzed by High-Performance Thin-Layer Chromatography Hyphenated with Effect-Directed Assays
Source: Molecules. 2021 Dec 19;26(24):7683. doi: 10.3390/molecules26247683 (PMC8709286; doi:10.3390/molecules26247683)
Supplement: Supplementary file 1 [file molecules-26-07683-s001.zip › molecules-1514637-supplementary.pdf]

Supplementary Materials

Puree and juice of Thai mango and pineapple analyzed by high-performance thin-layer chromatography hyphenated with effect-directed assays

G.E. Morlock,[a,ƚ], N. Wutthinithisanand[a,b,ƚ], D. Rauhut[b]*

[a]Chair of Food Science, Institute of Nutritional Science, and Interdisciplinary Research Center, Justus Liebig University Giessen, Heinrich-Buff-Ring 26-32, 35392 Giessen, Germany

[b]Department of Microbiology and Biochemistry, Hochschule Geisenheim University, Von-Lade-Str. 1, 65366 Geisenheim, Germany

[ƚ]Both authors contributed equally.

*Corresponding author. Tel. +49-641-99-39141. Fax +49-641-99-39149. E-mail address: Gertrud.Morlock@uni-giessen.de (G.E. Morlock).

Table S1. Investigated mobile phases 1–6, which led to successful separations in previous projects: methanolic extracts (10 µL; 0.4 g/mL or 4 mg/area) applied as 6 (or 7) x 3 mm area, developed on HPTLC silica gel 60 F_{254} plates with mobile phases as listed up to 60 mm, derivatized with anisaldehyde sulfuric acid reagent and documented at FLD 366 nm.

**1**	**2**
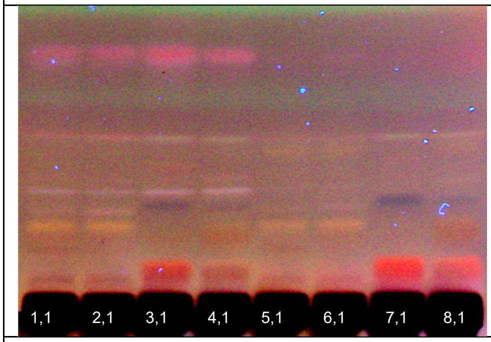	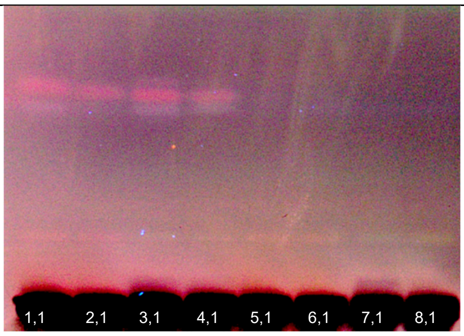
Toluene – ethyl acetate – methanol – formic acid 2.4:1.8:0.7:0.06, *V/V/V/V*	Toluene – ethyl acetate – formic acid 7:3:0.1, *V/V/V*

**3**	**4**	**5**	**6**
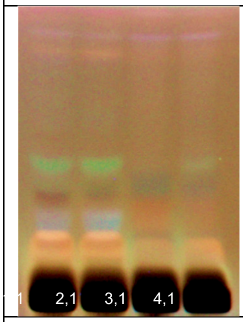	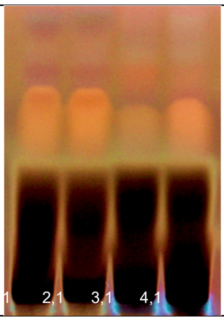	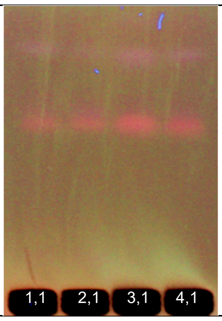	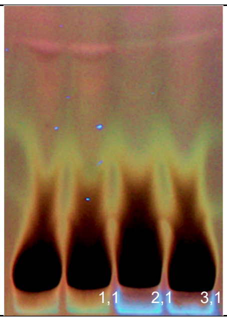
Ethyl acetate – toluene – formic acid – H_2O 8:2:1.5:1, *V/V/V/V*	Ethyl acetate – 2-butanone – formic acid – H_2O 5:3:2:1, *V/V/V/V*	*n*-Hexane – ethyl acetate 6.5:3.5, *V/V*	Acetonitrile – H_2O 4:1, *V/V*, + 8 mg diphenylboric acid - 2-aminoethylester

Table S2. Chromatograms at UV 254 nm and FLD 366 nm before the respective assay detection as in Fig. 4.

**UV 254 nm**	**FLD 366 nm**
Before the DPPH$^{\bullet}$ assay	
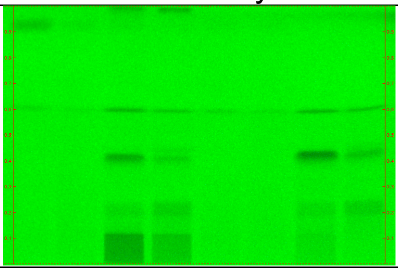	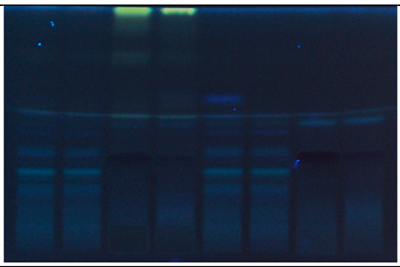
Before the *A. fischeri* bioassay	
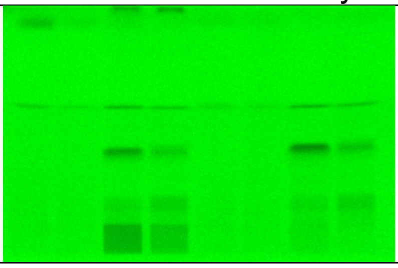	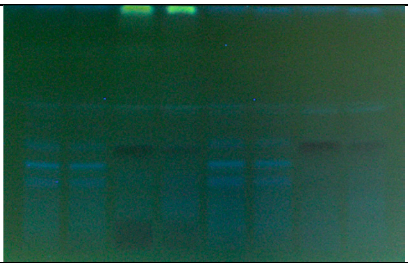
Before the *B. subtilis* bioassay	
No F_{254} plate	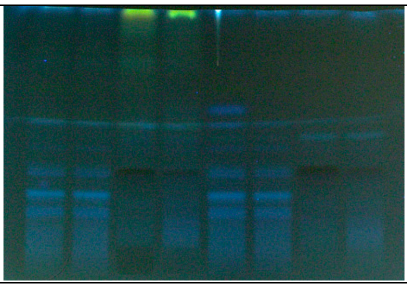
Before the AChE assay	
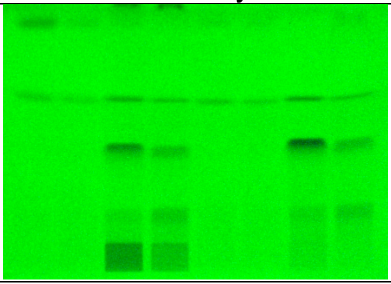	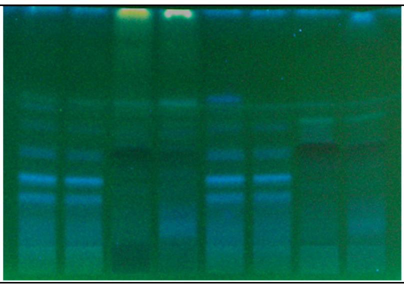
Before the tyrosinase assay	
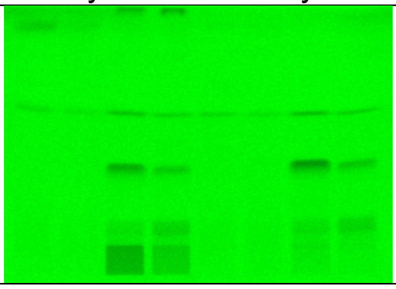	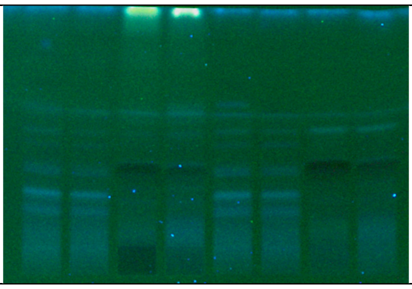
